# An initial map of chromosomal segmental copy number variations in the chicken

**DOI:** 10.1186/1471-2164-11-351

**Published:** 2010-06-03

**Authors:** Xiaofei Wang, Samuel Nahashon, Tromondae K Feaster, Ann Bohannon-Stewart, Nathaniel Adefope

**Affiliations:** 1 Department of Biological Sciences, Tennessee State University, 3500 John A. Merritt Blvd, Nashville, TN 37209, USA; 2 Department of Agricultural Sciences, Tennessee State University, 3500 John A. Merritt Blvd, Nashville, TN 37209, USA

## Abstract

**Background:**

Chromosomal segmental copy number variation (CNV) has been recently recognized as a very important source of genetic variability. Some CNV loci involve genes or conserved regulatory elements. Compelling evidence indicates that CNVs impact genome functions. The chicken is a very important farm animal species which has also served as a model for biological and biomedical research for hundreds of years. A map of CNVs in chickens could facilitate the identification of chromosomal regions that segregate for important agricultural and disease phenotypes.

**Results:**

Ninety six CNVs were identified in three lines of chickens (Cornish Rock broiler, Leghorn and Rhode Island Red) using whole genome tiling array. These CNVs encompass 16 Mb (1.3%) of the chicken genome. Twenty six CNVs were found in two or more animals. Whereas most small sized CNVs reside in none coding sequences, larger CNV regions involve genes (for example prolactin receptor, aldose reductase and zinc finger proteins). These results suggest that chicken CNVs potentially affect agricultural or disease related traits.

**Conclusion:**

An initial map of CNVs for the chicken has been described. Although chicken genome is approximately one third the size of a typical mammalian genome, the pattern of chicken CNVs is similar to that of mammals. The number of CNVs detected per individual was also similar to that found in dogs, mice, rats and macaques. A map of chicken CNVs provides new information on genetic variations for the understanding of important agricultural traits and disease.

## Background

Genomic variations within a species may involve changes as small as a single nucleotide to as large as microscopically visible chromosome segments, even whole sets of chromosomes. While microscopic genome variations were studied in cytogenetic laboratories for a long time, the readily availability of DNA sequencing technology and high throughput approach have popularized analysis on single nucleotide polymorphism (SNP) and microsatellites. It was not until recently that genome variation involving intermediate DNA segments, called segmental copy number variation (CNV), was recognized. This type of genome variation involves submicroscopic insertion, deletion, segmental duplication and complex changes of greater than 1 kb to several Mb in size [1-3]. Whole genome scanning studies for CNV have been conducted extensively in humans [4-10], chimpanzees [11,12], dogs [13,14], mice [15-18], rats [19] and swine [20]. Although several chicken CNV loci have been studied in a case-by-case manner [21,22], CNV in birds has received little attention. To our understanding, few publications are available describing whole genome CNV studies in birds [23].

Since its domestication 8,000 years ago, the chicken has provided table eggs, meat and ritual values to human society. Over the last 100 years, the chicken has also served as a model organism for fundamental biological and biomedical studies [24]. The first examples of oncogene and viral induced tumor were demonstrated in the chicken [25]. The B-lymphocytes were first identified in chickens. Spontaneous chicken mutants, such as the dwarf [26,27] and the retina degeneration [28], have provided rich information regarding particular gene functions. The regulation of chicken ovalbumin expression was studied extensively to elucidate the mechanism of eukaryotic transcription control [29] and steroid hormone actions [30,31]. Because of the historical, biomedical and agricultural importance, evolutionary distance and readily availability, the chicken is the leading species among farm animals in the development of genomics tools and resources, including the chicken genome assembly [32], genetic variation map of single nucleotide polymorphism [24,33], collections of comprehensive expressed sequence tags (EST) [34-37] and DNA microarray [38-40].

Distinct from mammalian genomes, typical avian genomes are composed of several large chromosomes and a group of microchromosomes that are indistinguishable microscopically with conventional karyotyping techniques [41,42]. The chicken genome has 1.2 billion base pairs on 39 pairs of chromosomes, including a pair of sex chromosomes ZZ for males and ZW for females [43,44]. Despite that the chicken genome is one third of a typical mammalian genome in DNA content, it was predicted to have a similar number of genes [37]. Thus, it would be conceivable that the chicken has reduced intergenic spaces and reduced repetitive sequence content. How the compacted genomes vary in chromosomal segmental copy number and how these variations affect important agricultural and biomedical traits are of great interest. Here we provide a snapshot of CVNs in the chicken genome.

## Results and Discussion

### Mapping of CNVs in chickens

NimbleGen whole genome tiling arrays containing 385,000 probes were used to analyze chicken CNVs. Four broilers (Cornish Rock, 2 males and 2 females), four Leghorns (2 males and 2 females) and two Rhode Island Reds (males) were analyzed with array comparative genome hybridization (aCGH). One additional male broiler DNA was used as a reference for all hybridizations. CNVs were identified by comparing ratio between the test and the reference and all CNV loci were visually inspected on aCGH data plots (Additional file 1, Fig. S1). We identified 96 high confidence or suggestive CNVs. When CNV signals in two or more animals overlapped on a chromosome, they were considered to be high confidence CNV. On average, seventeen CNVs were called in each bird.

These CNVs were found on chicken chromosomes (GGA) 1-8, 10-18, 20, 22-27, and Z (Table 1 and Additional file 2, Table S1). Due to poor probe coverage, data on W chromosome were removed from analysis. The 96 CNVs encompassed 16 Mb, which is about 1.34% of the entire chicken genome. Among the 96 CNV loci, forty six loci were non-coding sequences (5.1 Mb).

**Table 1:** High confidence CNVs in chickens

***Locus ID***	***Chromosome***	Start position*	***Size (bp)***	***Gene***	***Status^#^***			***Number of observations***
						
					Broiler	Leghorn	RIR^†^	
1	1	4015022	47654	LOC419112	loss	loss	loss	6
2	1	44980061	80399	Non-coding	loss	loss		7
3	1	48005486	34931	Non-coding	gain			3
4	1	59885173	32693	CHRM2		loss		2
5	1	165880299	112347	ZFR, IL-3	gain	gain		4
6	2	40647961	39933	Spliced ESTs		gain	loss	2
7	2	95665092	10297	Non-coding	gain	gain		4
8	2	97295434	44839	Non-coding	gain	gain		5
9	2	134727846	102330	MGC24975(SZD6)	gain	gain		3
10	2	154562776	299829	SCRIB	loss	gain		2
11	3	113612615	40053	MRPL19,	loss		loss	2
12	4	62172811	12364	Non-coding	loss		loss	2
13	4	88897639	175343	RHACD8	gain	loss	loss	5
14	4	89602897	42219	Non-coding		loss		3
15	5	22120222	92556	Non-coding		loss		3
16	6	12150334	84982	Non-coding		loss	loss	3
17	10	125113	32851	olfactory receptor 6k2	gain	gain		2
18	11	2670295	14897	Non-coding	gain			3
19	12	15208	77317	Non-coding	loss			2
20	13	2722503	60308	EST	loss		loss	3
21	16	200114	12820	Zinc finger protein	loss	loss		2
22	16	270019	162832	HLA class I antigen	loss	loss		2
23	17	567532	57890	PRF1	gain			2
24	Z	9965426	192201	PRLR	loss	loss		2
25	Z	71975037	142814	CDKN2A, MTAP	loss	loss	loss	3
26	Z	73495364	29964	Non-coding	gain	gain		2

Note: *, the start position of the CNV was based on 2006 chicken genome assembly. #, gain or loss was assigned based on common reference DNA from one broiler bird (#6281). †, Rhode Island Red.

There were 26 high confidence CNVs that were observed at least in two birds (Table 1). Among these high confidence loci, eleven loci involve non-coding sequence only, which sum up to 525 kb. The remaining 15 loci occupied about 1.5 Mb, involving one or more coding sequences.

Variations at locus 13 (chr4:88,897,639-89,072,982) on GGA4 (Table 1) appear to be complex. When compared with the reference, three birds showed loss of 150-170 kb, while two other birds showed a gain of 112 kb and 172 kb. Locus 10 on GGA2 has three clearly different alleles, one of which was a gain of 300 kb and the other was a loss of 20 kb region and the third allele was without gain or lost. However, visual inspection of corresponding aCGH plots revealed that several other samples may also have copy gains (Additional file 3, Fig. S2 A).

In our dataset, two CNV calls were made for chromosome 25. One of the calls appears to be a gain of an entire chromosome 25 in bird #5849 (Additional file 2, Table S1), but no obvious visual abnormality of the bird was observed. The other CNV involves the first 10-kb region of the chromosome assembly.

The majority of the high confidence CNVs was shared across breeds, suggesting their relative "ancient" origin. Some high confidence CNVs were specific to individual breeds. Whether they are breed-specific requires further evaluation of a much larger sample size. It is not clear whether these putative private CNVs contribute to breed specific biology. Fewer high confidence CNVs were found in Rhode Island Reds when compared with other strains evaluated. This may be attributable to the small number of animals analyzed, small founder population and/or population diversity.

Seventy CNVs, which encompassed 14 Mb, were observed only once in our data set (Additional file 2, Table S1). Of the seventy CNVs, twenty seven CNVs involved only non-coding sequences (1.8 Mb). Twenty nine CNVs showed loss of DNA while the rest showed gains of DNA. The majority (62%) of CNVs with DNA loss were non-coding sequences. In contrast, the majority (80.5%) of CNVs with gain of DNA involve coding sequences. In addition, sizes in CNVs of DNA loss tend to be smaller (mean 43.2 kb and median 30 kb) compared with that of DNA gain (mean 313 kb and median 67 kb).

Although probes assigned to unknown chromosome locations were excluded from CNV calls, an ambiguous segment on GGA 20 (chr20_random in chicken genome draft 2.1 at UCSC genome database) is noteworthy. The entire segment was 72 kb according to the genome assembly. Several ESTs and mRNAs were mapped to this segment. Data from aCGH strongly support that this region be assigned to W chromosome [45]: All female birds showed gain of copies with high scores, when compared with the reference.

### Quantitative PCR analysis and CNV validation

**Figure 1:**
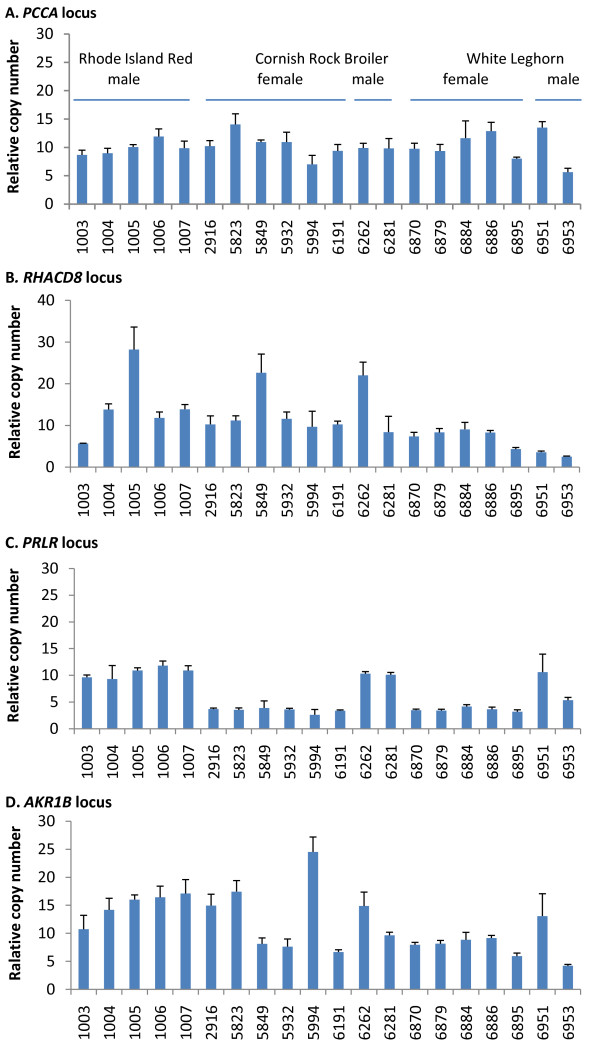
**Quantitative PCR analysis of CNVs in 20 birds**. DNA samples were diluted to 10 ng/μl and the conecentrations were meassured with Nanodrop spectrophotometer. Relative copy number was obtained by comparing threshold cyles of test DNA with a reference DNA that was serial diluted to 80, 20, 10 and 2.5 ng/μl. One unit of relative copies is the amount in 1 ng of reference DNA. Values represent mean ± SD of four reactions. Data were not normalized to the reference locus and copy number was not rounded. Numbers on x-axis are bird ID.

Real time quantitative PCR (qPCR) was performed to validate aCGH data at five loci. Two of the five loci (e. g. *PCCA*, *THRSP*) served as references of no variation in copy number, while three loci were CNV detected with aCGH. The *PCCA *locus encodes propionyl coenzyme A carboxylase. Analysis of chicken genome assembly indicated that a single copy of this gene exists in a haploid genome. Two copies of *THRSP *genes exist in chickens [46]. The qPCR results for *PCCA *locus showed minimal variations among 20 birds (including 11 birds examined with aCGH). We attribute these variations to random errors, including DNA dilution error. Similar qPCR results were obtained for *THRSP *locus in 23 birds (including 20 birds examined for PCCA), which also showed minimal variations among birds except that two birds appeared to have lost copies (data not shown). Three CNV loci (e. g. locus 13:*CD8α-RHACD*, locus 24:*PRLR*, and a suggestive locus *AKR1B*) were examined twice: once estimated with standard curve method (Fig. 1B,C,D) in 20 birds and once with 2^-ΔCt ^method in 23 birds (not shown). Results of the two separate qPCR assays were concordant. F-tests were performed to determine whether copy numbers detected with qPCR have the same variance between the reference locus and CNV loci. Results indicate that all three CNV loci had greater variance than the references (*P *< 0.05 for *PRLR *locus and *P *< 0.01 for *AKR1B *and *RHACD8*), suggesting the three loci were truly CNV.

Locus 13 involves *RHACD8*, which was reported to have variable copy numbers among different breeds of chickens. Our qPCR data indicated relative copy number of *RHACD8 *locus was highly variable among chickens. Birds with the highest copy number had seven times as much as those with the lowest copy number.

Locus 24 involves prolactin receptor gene (*PRLR*). In our aCGH assay, this locus was identified in 2 females. Another bird appeared to be false negative, because visual inspection of aCGH plots revealed likely shifting of the log2 ratio (Additional file 3, Fig. S2 B). Subsequent qPCR analysis showed that all female birds have a single copy, and males showed 3 or 4 copies. Since *PRLR *is located on GGA Z, males are expected to have two copies per cell, whereas females have one copy should there be no CNV. However, χ^2 ^tests indicated that the relative copy number ratio were 3:1 between males and females (Fig. 1C). It appeared that the male chicken # 6953 did not have three folds the copy number of females. However, after DNA concentration correction, its relative copy number was the same as other males. Results of the separate qPCR assay with 2^-ΔCt ^method agreed with this notion. The sex specific CNV at this locus can be explained by the industrial practice, as recently reported by Elferink [47]. A sex-linked late feathering allele *K *containing 2 copies of *PRLR *has been introduced to commercial flocks and used widely for sexing hatchlings. This *K *allele is incomplete dominant to the early feathering *k^+ ^*allele containing one copy of *PRLR*. One of our bird suppliers crosses *k*^+^*k*^+ ^males with *K*W females, such that progeny females are early feathering *k*^+^W containing one copy of *PRLR*, while the male progeny are late feathering *Kk*^+ ^containing three copies of *PRLR*.

The CNV locus on GGA 1, involving aldo-keto reductase 1B (Additional file 1, Table S1, *AKR1B1 *locus, chr1:64280187-64310165), was first identified as a suggestive CNV found in only one bird with a gain of copy. A qPCR assay showed that the variation in copy number was far more frequent (Fig. 1D) than it appeared in the aCGH: All Leghorn chickens had the least copies (presumably two copies) and all Rhode Island Reds doubled that figure, while Cornish Rock broilers have variable numbers from 2 to 7 copies. Visual inspection of this locus in aCGH plots did not reveal convincingly significant variations.

### Complexity of chicken CNVs

**Figure 2:**
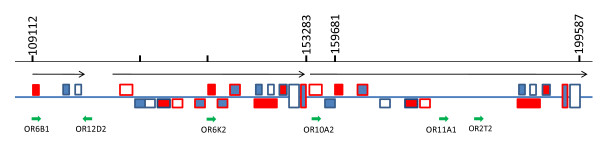
**Organization of CNV region at locus 17 located on GGA 10 (Chr10: 125113-157964)**. A block of sequence (Chr10:125051-149999) was aligned with chicken genome assembly build 2.1 using BLAT algorithm at UCSC genome database. Numbers at the top of the graph represent nucleotide positions in the chicken genome assembly. Long arrows indicate higher order repeat organization and orientation. Short green arrows indicate orientation and location of human olfactory receptor homologs aligned to GGA 10. Symbols of the same style (line color and fillings) on the same side of the blue line represent sequence blocks sharing >95% identity.

In order to understand genomic organizations of DNA sequences involved in CNV, we mapped DNA sequences that are similar to the ones in CNV regions by BLAT search. This mapping revealed the organization complexity of some CNVs. For example, according to chicken genome build 2.1, locus 17 on GGA 10 showed duplication of various blocks in a 90-kb region (Fig. 2). This locus contains at least 6 copies olfactory receptor-like sequences, organized in grossly three larger repeating units in the same orientation. Blocks of several hundred to several thousand base pairs are highly conserved (>95% identity in nucleotide sequences) among these repeating units. However, the relative positions of these blocks were shuffled to different places.

The *CD8α *locus on GGA 4 is also organized in a complex way. Although it is known to be polymorphic among breeds [48], we did not anticipate such complex and extensive variations as revealed by qPCR. Other loci examined also show more or less complexity.

### Functional implications of chicken CNVs

Among the high confidence CNV loci, at least 15 loci involve partial or entire functional genes. Many of these functional genes have paralogs in the chicken genome. For example, CNV locus 5 on GGA 1 (Chr1: 165880299-166002720) encodes a zinc finger RNA binding protein (ZFR). In the chicken, a second copy of *ZFR *is located on Z-chromosome (chrZ: 9,104,112-9,144,261). Locus 17 (chr10: 125113-157964) involves an olfactory receptor 6K1 (OR6K1)-like sequence.

The CNV at *CHRM2 *locus (locus 4) was observed in two of the Leghorn birds. *CHRM2 *is one of the five muscarinic acetylcholine receptor genes that play important roles in numerous physiological functions including higher cognitive processes such as memory and learning [49]. EST and other sequence data indicate that the chicken *CHRM1*, *4 *and *5 *are located on chromosome 5, while *CHRM2 *and *3 *are mapped to chromosomes 1 and 3 respectively. Our aCGH data suggest the loss of *CHRM2 *copies in Leghorn chickens. Analysis of chicken whole genome assembly has not found any evidence that *CHRM2 *locus involves a recent duplication. Thus, deletion of *CHRM2 *is more likely the scenario in the Leghorn birds.

According to the May 2006 chicken (*Gallus gallus*) v2.1 draft assembly, the chicken *AKR1B *locus contains 4 consecutive copies of *AKR1B *organized head-to-tail. Although all copies appear to be transcribed since ESTs were found for all of them, the telomere-proximal copy appears to be more actively transcribed, as evidenced by the greater number of ESTs found for this copy. The four *AKR1B *copies share 80-92% amino acid residues. However, the telomere-proximal two copies are less similar from each other as well as from the two centromere-proximal copies in intron sequences. The two centromere-proximal genes contain large blocks of sequences similar to each other, including introns. In vertebrates, each species has several *AKR1Bs *that are expressed in most tissues [50]. The AKR 1B subfamily catalyzes the reduction of aldehydes [51]. Members of aldose reductase (AKR1B7, AKR1B10) may regulate fatty acid synthesis [52,53].

**Figure 3:**
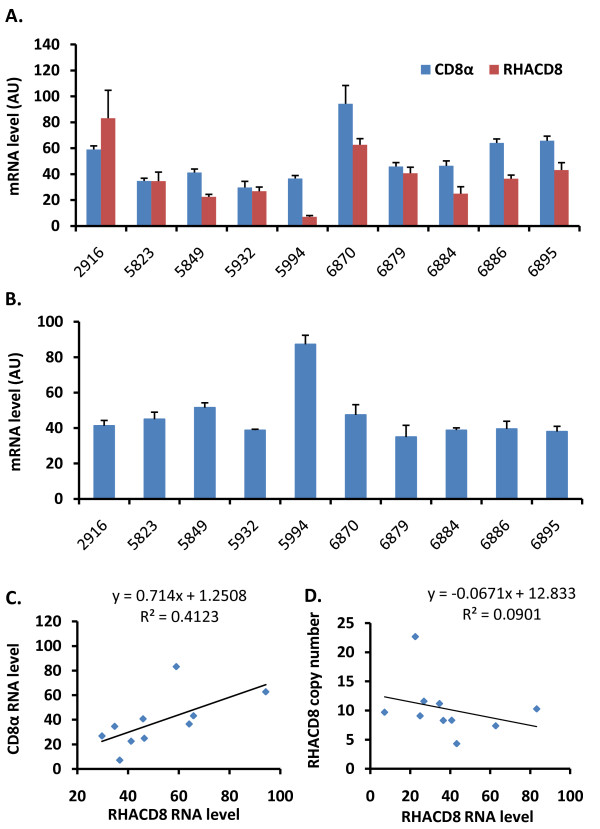
**Relationship between copy number and transcript levels at *RHACD8 *locus**. (A) Plot of spleen *CD8α *and *RHACD8 *mRNA levels. Spleen RNA samples were isolated from adult broilers and Leghorns. (B) Plot of GAPDH mRNA levels in spleen of the same 10 chickens. (C) Pearson correlation between *CD8α *and *RHACD8 *mRNA levels. (C) Pearson correlation between *RHACD8 *mRNA level and of *RHACD8 *DNA copy number.

An EST was found to be derived from CNV locus 13. The sequence have been predicted to be a CD8α- like messenger RNA -*RHACD8*, which was shown to be expressed in spleen [48]. We seek to determine whether the copy number variation could affect the level of *RHACD8 *transcript. Spleen RNA levels of *RHACD8 *and *CD8α *were examined in five broiler and five Leghorn females with RT-qPCR in two separate experiments (one with standard curve method and one with 2^-ΔCt ^method, Fig. 3). RNA levels determined with 2^-ΔCt ^method were highly correlated with those determined with standard curve method (r = 0.88 for *RHACD8 *and r = 0.96 for *CD8α*). Glyceraldehyde 3-phosphate dehydrogenase (GAPDH) and β-actin mRNAs were examined as controls. Levels of β-actin mRNA varied remarkably among these chickens (data not shown). GAPDH mRNA levels were less variable (Fig. 3B), though bird # 5994 showed much higher level of *GAPDH *transcript. CD8α mRNA level appeared to be correlated with the level of *RHACD8 *transcripts (r = 0.64, or 0.56), at a marginal statistical significance (*P *= 0.045 or 0.08). But there was no evidence of correlation between levels of *RHACD8 *transcript and DNA copy number (*P *> 0.05, Fig. 3).

### Comparative genomics of chicken CNVs

Since the platforms for CNV detection vary from species to species, much of the information cannot be compared directly across species. However, similarities can be found among studies using species-specific NimbleGen tiling arrays [13,16,19,54]. The NimbleGen mammalian arrays usually have a greater median probe space (5 kb) than the chicken array (2.6 kb). Accordingly, the average CNV sizes detected in mammals are larger than in chickens. However, the CNV calls in each individual are comparable among mouse, dog, and rhesus macaques [54]. Because the human genome was studied more extensively, up to 18.8% of human genome have been found in CNV regions [55]. Lower CNV coverage was found in other species. Our current data showed that chicken DNA sequences residing in CNV regions account for 1.34% of the genome, similar to that found in rat [19]. However, it is likely a significant underestimation of real CNVs in chickens, since a limited number of individuals have been surveyed. Furthermore, due to the incompleteness of the chicken genome assembly, a significant portion of the genome was not surveyed. The entire W chromosome was excluded from the analysis, and all probes that were assigned to ChrUn and chromosome-random were also excluded.

Out of thirteen chicken-turkey CNVs reported by Griffin et al, [23], three loci overlapped with our high confidence CNVs and four loci overlapped with our CNVs of single observation. These data suggest that some of our single observations are true CNVs. The overlapping loci have size disagreement between Griffin's and our study, possibly due to the use of different references. Red jungle fowl was used as the reference by Griffins *et al*, while a Cornish Rock broiler bird was used as the reference in our study. The discrepancy may be attributable to non-recurrent rearrangement. Apparently, larger populations need be examined to obtain a comprehensive picture of chicken CNV.

It is conceivable that lost DNA segments tend to be small in size and non-coding, while large CNV regions tend to emerge from gain of DNA and involve more functional genes, because large segment loss may be detrimental when these alleles are homozygous. Similar observations were also found in other species [2,56]. A significant number of CNVs involves members of paralogs. This can be explained by the fact that paralogous genes may compensate for lost copies. Similarly, a significant enrichment of CNVs in segmental duplications was found in the mouse [18].

It appeared that aCGH method tends to report larger segments being involved in the duplication/deletion than they really are. For example, the aCGH reported the involvement of 190 kb in the duplication of the *K *locus on Z chromosome. Detailed studies by Elferink et al [47] showed this duplication involves only 176 kb. This discrepancy is consistent with recent report that most CNVs are smaller in size than revealed by larger probe spacing [57].

It is of major interest to map the impact of CNVs in relation to disease, immunity, and agricultural traits. It has been shown that some CNVs contribute to phenotypic variations while others are amenable for genome-wide association study for their influence on genetic disease or disease susceptibility. Nevertheless, large amount of putatively functional sequences, including protein coding sequences and conserved non-coding sequences, fall within or flank CNVs. In humans, although most CNVs were detected in apparently "healthy" individuals, many CNVs may have subtle, quantitative or late-onset phenotypic implications [58]. Functional attributes of the currently known CNVs are remarkably enriched in genes involved in environmental molecular interactions, including cytochrome p450 genes, immunoglobin-like receptors, defensins [59]. CNVs may affect phenotype by altering transcriptional level of genes within or adjacent to CNVR and subsequently alters translation levels. Such transcriptional and translational changes have already been demonstrated [60,61].

## Conclusion

The chicken genome was examined for chromosomal segmental copy number variations with whole genome tiling arrays. Twenty six high confidence CNVs that were observed in two or more birds and seventy CNVs that were observed once were identified. The majority of the high confidence CNVs was shared across breeds (broiler, Leghorn, and Rhode Island Red). Fifteen CNV loci involve functional genes, or spliced EST coding sequences. Although CNVs that were observed once require further confirmation, some of them represent true CNVs.

The mapping of CNVs in chicken could provide new opportunity for understanding genomic variation and related phenotypic characteristics. This mapping will also contribute to association studies in effort to map traits of economic importance.

## Methods

DNA samples: Blood samples were collected from 3 strains of chickens (Cornish Rock broiler, Leghorn and Rhode Island Red) with 0.5 M EDTA and stored at -20°C until DNA isolation. Leghorn and broiler birds (commercial generation) were purchased from Ideal Poultry (Texas, USA). Rhode Island Red birds were purchased from Murray McMurray Hatchery (Iowa, USA). DNA was isolated with DNeasy genomic DNA isolation kit or phenol chloroform extraction. All DNA samples for array hybridization were analyzed with agarose gel electrophoresis and spectrophotometry. DNA concentrations were measured with NanoDrop spectrophotometer (NanoDrop Technologies, Willmington, DE). Ten samples (4 Leghorns, 4 broilers and 2 Rhode Island Reds) were analyzed with array CGH and twenty three samples were analyzed by qPCR. In addition, a broiler male was used as the reference for all aCGH analysis. The use of animals was approved by Tennessee State University Institutional Animal Care and Use Committee (IACUC).

Hybridization: ACGH was carried out using whole genome tiling array galGal3_WG_CGH. This array platform was designed from the chicken genome build 2.1 from UCSC genome database (2006). The array contained 385,000 probes of 50-75mer. The mean probe spacing was 2557 bp and the median probe spacing was 2586 bp.

Each test DNA sample, labeled with Cy3, was co-hybridized with the reference male broiler sample (labeled with Cy5). The hybridization and initial data analysis (normalization and segmentation) were performed by NimbleGen Systems Inc (Madison, WI, USA). Segmentation analysis was performed with NimbleScan 2.4 software (segMNT algorithm). NimbleGen has provided literature package describing the technical specifics http://www.nimblegen.com/products/lit/lit.html. Criteria for CNV calls were similar to Chen *et al *[13] and Graubert *et al*. [16]. Segments of five or more probes with mean log_2 _ratio shift from baseline greater than +/- 0.3 were flagged as candidate CNV. Probes from uncertain chromosomal loci (Chr#-random and ChrUn-random in the UCSC database) and from W chromosome were removed from the results. Raw aCGH data for this study have been deposited to GenBank GEO database under accession GSE19469 http://www.ncbi.nlm.nih.gov/geo/query/acc.cgi?acc=GSE19469.

QPCR: PCR primers were designed using Primer Express 2.0 (Applied Biosystems, Carlsbad, CA), Sequences of the primers are available in Additional file 4, Table S2. All qPCR assays were conducted using SYBR GreenER qPCR kit (Invitrogen, Carlsbad, CA). Reaction was done in 20 μl containing 20 ng of genomic DNA (approximately 16,000 copies), 0.4 μM of each primer. Thermal cycles were: 1 cycle of pre-incubation at 50°C for 2 min and 95°C for 8 min, 35 cycles of amplification (95°C for 15 s and 60°C for 60 s). Primers were validated by melting curve analysis, amplification analysis, standard curve, and no-template control reactions. For standard curve analysis, one DNA sample was serial diluted to 10, 20, 40 and 80 ng/μl, and measured again with spectrophotometer. Each concentration was analyzed in quadruplicates with qPCR to determine amplification efficiency. These assays showed amplification efficiencies between 112.4% and 132.7%, and correlation coefficients between 0.971 and 0.990. For melting curve analysis, PCR product of each primer set showed a single melting peak. Two separate qPCR assays were performed to determine relative copy numbers for each of three CNV loci (*RHACD8*, *PRLR*, and *AKR1B*). The first assay tested 23 birds and relative copy numbers were estimated with 2^-ΔCt ^method after primer validation. The second assay was done essentially the same way for 20 birds (included in the first assay) except that standard curve was generated concomitantly in the same plate with 4 concentrations (80, 20, 10 and 2.5 ng/μl) and copy numbers were estimated based on standard curve. Efficiencies of the second qPCR assay were 78.7%, 94.8%, 91.3% 95.7%, for *PCCA*, *RHACD8*, *PRLR *and *AKR1B *loci respectively.

Each test genomic DNA was diluted in Tris-EDTA (10 mM TRIS-HCl, 1 mM EDTA) buffer to 10 ng/μl, assessed with qPCR in quadruple reactions. QPCR was performed with iCycler (Bio-Rad, Hercules, CA), 96-well plate (Bio-Rad, cat# 2239441) and optical adhesive film (Applied Biosystems, Part # 4311971). To avoid potential uneven heating of reactions at the edge of thermal block, perimeter wells of PCR plates were routinely avoided when possible. In the first qPCR assay, copy numbers were assigned using the relative method 2^-ΔCt^, where ΔCt is the threshold cycle difference between the test sample and an arbitrarily selected reference sample that was used as a standard. The reference was considered to have two copies (when the standard is an autosomal locus) or one copy (when a locus is on Z chromosome of a female). In the second qPCR assay, relative copy numbers were assigned by comparing the Ct values with standard curve and the amount of copies in 1 ng of reference DNA (assumed as one unit).

RT-qPCR: Spleen of adult broiler and Leghorn was removed immediately after sacrificing birds by cervical dislocation, briefly frozen in liquid nitrogen, and transferred to -80°C until RNA isolation. Total RNA was extracted with RNeasy kit (Qiagen, Valencia, CA). RNA concentration was determined with NanDrop and diluted to 25 ng/μl for transcript level analysis. An EST (GenBank accession CF255001) derived from CNV locus 13 (chr4:88,954,181-88,987,642) was used to design primers for transcript level analysis. RT-qPCR was carried out as described previously [62] with slight modifications using QuantiTect SYBR Green RT-PCR kit (Qiagen). To use iCycler equipment with the RT-PCR kit, fluorescein (Bio-Rad) was added to a final concentration of 10 nM. Equipment and plastics were the same as used in QPCR. Each reaction was carried out in 20-μl volume containing 50 ng of total RNA and 0.4 μM of forward and reverse primers. Reverse transcription was done at 50°C for 10 min, followed by 1 cycle of incubation at 90°C for 15 min, and then 35 cycles of amplification (95°C for 15 s and 60°C for 60 s). No amplification product was seen in no-template control reactions. Threshold cycles for no-reverse transcriptase control were at least 7 cycles greater than that for reactions with reverse transcriptase. Similar to qPCR assay on DNA, two separate RT-qPCR assays were conducted for *CD8α *and *RHACD8*: the first one with 2^-ΔCt ^method and the second one with standard curve method. RT-qPCR efficiencies for *CD8α *and *RHACD8 *primers, obtained by at least three reproducible standard curve analyses, were 96.3% ~ 97.5% and 80.1% ~ 106.2%, respectively, all with correlation coefficient > 0.99. The second RT-qPCR assay was performed for *GAPDH*, β-actin *CD8α *and *RHACD8*, concomitantly with standard curve. Efficiencies were 102.1%, 127.2%, 85.7% and 83.9% respectively. Levels of transcripts were expressed relative to the amount in 1 ng of total RNA in the reference sample.

## Abbreviations

aCGH, array comparative genome hybridization; AKR1B, aldo-keto reductase 1B; CD8α, CD8 antigen alpha chain; aCGH, array comparative genome hybridization; CHRM, cholinergic receptor muscarinic; CNV, copy number variation; CNVR, CNV region; EST, expressed sequence tag; GAPDH, glyceraldehyde 3-phosphate dehydrogenase; Mb, million base pair; PCCA, propionyl coenzyme A carboxylase; PCR, polymerase chain reaction; PRLR, prolactin receptor; qPCR, quantitative real time PCR; RT-qPCR, quantitative real time reverse transcriptase-PCR; SNP, single nucleotide polymorphism; THRSP, thyroid hormone responsive spot 14; ZFR, zinc finger RNA binding protein.

## Authors' contributions

XW conceived, designed and performed experiment, analyzed data, wrote the manuscript. SN conceived, designed and performed experiment, analyzed data, wrote the manuscript. TKF performed DNA isolation, qPCR analysis and discussed manuscript. AB performed qPCR, RT-qPCR assay. NA managed animals and collected samples and discussed the manuscript. All authors read and approved the final manuscript, except NA, who passed away during the review.

## Supplementary Material

Additional file 1 **Fig. S1:** Examples of aCGH plot for 26 high confidence CNV. Click here for file

Additional file 2 **Table S1:** CNV loci observed once. Click here for file

Additional file 3 **Fig. S2:** Examples of likely false negative CNV occurrence. Click here for file

Additional file 4 **Table S2:** Primers used in the study. Click here for file
