# Supplementary material for: An initial map of chromosomal segmental copy number variations in the chicken
Source: BMC Genomics. 2010 Jun 3;11:351. doi: 10.1186/1471-2164-11-351 (PMC2996973; doi:10.1186/1471-2164-11-351)
Supplement: Additional file 1 — Fig. S1: Examples of aCGH plot for 26 high confidence CNV. [file 1471-2164-11-351-S1.DOC]

**Fig. S1. aCGH signal plot of high confidence CNV loci**

X-axis is the nucleotide position on chromosome shown on top of each panel. Y-axis is the log_2_ ratio of fluorescent intensity from aCGH. Letters at the end of the bird # indicate breed (R, Rhode Island Red; B, Cornish Rock broiler; L, Single Comb White Leghorn) and gender (M, Male; F, Female). Horizontal red lines indicate segmental mean Log_2_ ratio (note that some mean ratios on GGA 1 were not stably displayed in the plot due to software limitation).


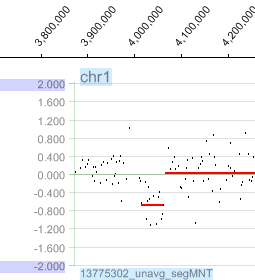

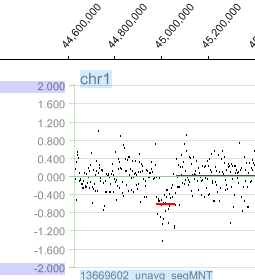


**Locus 1:** bird # 1007_RM **Locus 2:** bird # 5849_BF


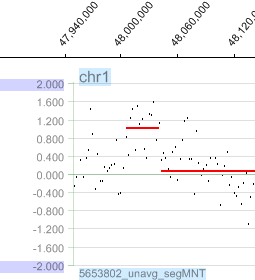

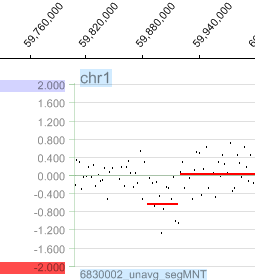


**Locus 3:** bird # 6191_BF **Locus 4:** bird # 6951_LM


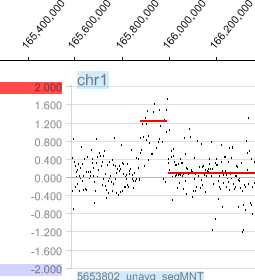

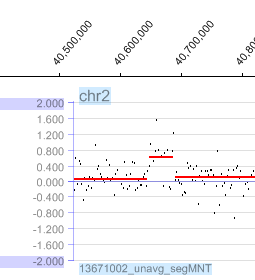


**Locus 5:** bird # 6191_BF **Locus 6:** bird # 6884_LF


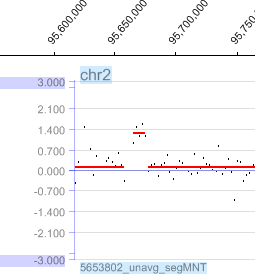

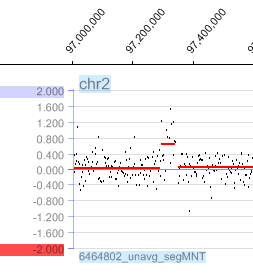


**Locus 7:** bird # 6191_BF **Locus 8:** bird # 2916_BF


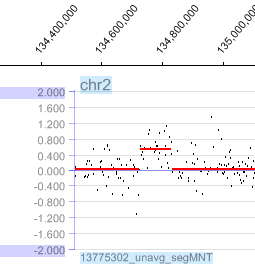

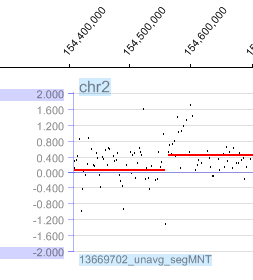


**Locus 9:** bird # 1007_RM **Locus 10:** bird # 6870_LF


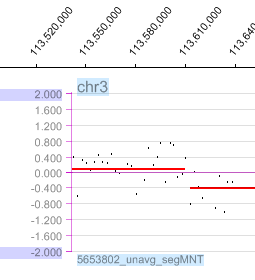

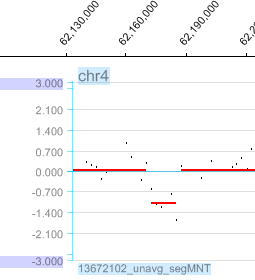


**Locus 11:** bird # 6191_BF **Locus 12:** bird # 1003_RM


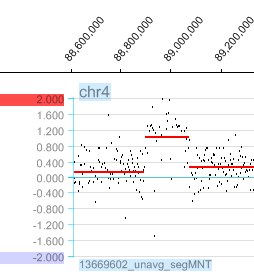

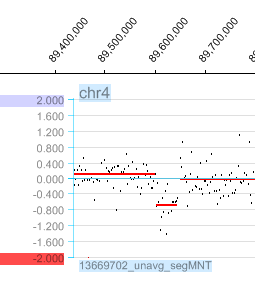


**Locus 13:** bird # 5849_BF **Locus 14:** bird # 6870_LF


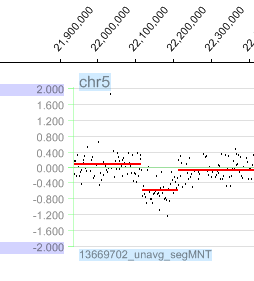

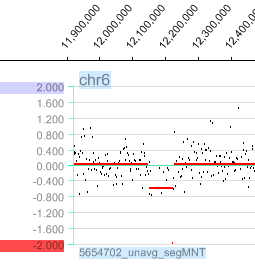


**Locus 15:** bird # 6870_LF **Locus 16:** bird # 6953_LM


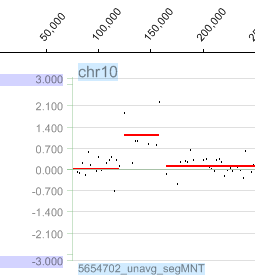

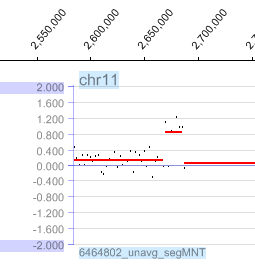


**Locus 17:** bird # 6953_LM **Locus 18:** bird # 2916_BF


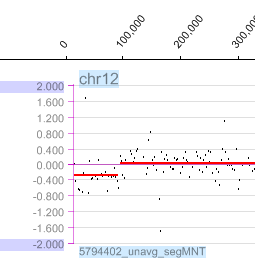

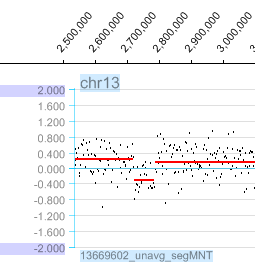


**Locus 19:** bird # 6262_BM **Locus 20:** bird # 5849_BF


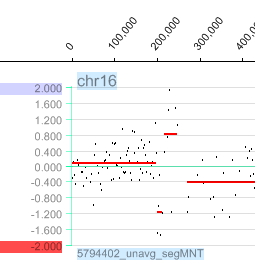

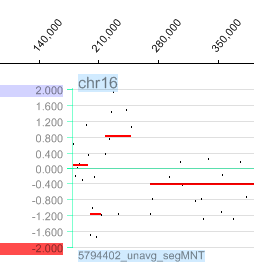


**Locus 21:** bird # 6262_BM **Locus 22:** bird # 6262_BM


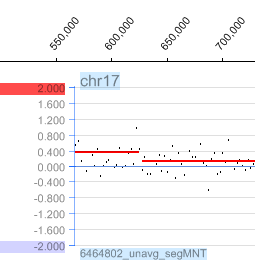

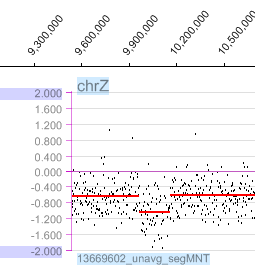


**Locus 23:** bird # 2916_BF **Locus 24:** bird # 5849_BF


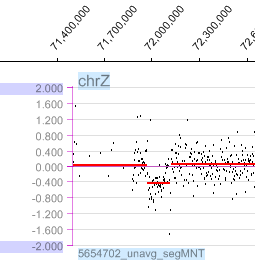

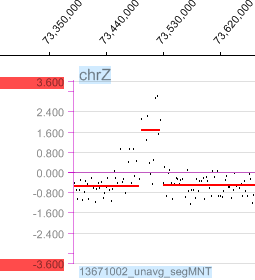


**Locus 25:** bird # 6953_LM **Locus 26:** bird # 6884_LF
