# Supplementary material for: An initial map of chromosomal segmental copy number variations in the chicken
Source: BMC Genomics. 2010 Jun 3;11:351. doi: 10.1186/1471-2164-11-351 (PMC2996973; doi:10.1186/1471-2164-11-351)
Supplement: Additional file 3 — Fig. S2: Examples of likely false negative CNV occurrence. [file 1471-2164-11-351-S3.DOCX]

**Fig. S2. Example of likely false negative CNV occurrence (arrow head) on GGA 2 (A) and Z (B).** We noted that the significant baseline shift in four samples (13669602, 13669702,13671002 and 5653802) in B was due to female test DNA was hybridized against male reference DNA.
